# Effect of concomitant administration of coenzyme Q10 with sitagliptin on experimentally induced diabetic nephropathy in rats

**DOI:** 10.1080/0886022X.2016.1254659

**Published:** 2016-11-13

**Authors:** Rajesh Maheshwari, Ramachandran Balaraman, Ashim Kumar Sen, Disha Shukla, Avinash Seth

**Affiliations:** Department of Pharmacy, Sumandeep Vidyapeeth, Piparia, Vadodara, Gujarat, India

**Keywords:** Streptozotocin, diabetic nephropathy, coenzyme Q10, sitagliptin, TNF-α, TGF-β

## Abstract

This study was aimed to investigate the therapeutic potential of coenzyme Q10 and its combination with sitagliptin in experimentally induced diabetic nephropathy. The diabetic rats were treated with coenzyme Q10 or sitagliptin and their concomitant administration. Various parameters of renal function like serum creatinine, urea, uric acid and markers of oxidative stress such as renal malondialdehyde content (MDA), glutathione (GSH) level and superoxide dismutase (SOD), catalase activities were measured. TNF-α, TGF-β, MPO activity and nitrite content were estimated in renal tissue with histopathological observation. Diabetic rats showed a significant reduction in renal function, which was reflected with an increase in serum creatinine, urea and uric acid levels. Streptozotocin-nicotinamide caused renal tubular damage with a higher MDA level, depletion of SOD and CAT activity and GSH level. In addition, TNF-α, TGF- β, MPO activity and nitrite content were significantly increased in diabetic rats. Treatment with coenzyme Q10 or sitagliptin and their co-administration ameliorated STZ-nicotinamide-induced renal damage which was reflected by decreased oxidative stress, TNF-α, TGF-β, MPO activity, nitrite content along with histopathological changes. To conclude, concomitant administration of coenzyme Q10 and sitagliptin showed a better renoprotective effect than coenzyme Q10 or sitagliptin when given alone.

## Introduction

About more than 400 million people will be suffering from diabetes mellitus by 2030 and it is one of the world’s most common chronic metabolic disorders of multiple etiologies.^1^ This disease is the leading cause of morbidity and mortality because of its complication like macrovascular and microvascular disorders which lead to nephropathy and other cardiovascular diseases.^2^ End-stage renal failure is one of the consequences of diabetic nephropathy (DN).^3^ Proteinuria, an indicator of underlying DN, usually worsens with progression of diabetic kidney disease. Proteinuria and end stage renal failure are characterized by declining glomerular filtration rate and kidney structural changes, including thickening of the basement membranes and mesangial sclerosis. Hyperglycemia is a major cause of advanced glycation end products (AGEs) which is responsible for the cause of renal dysfunction in diabetes.^4^^,^^5^ It has also been found that oxidative stress is further responsible for the pathogenesis of diabetic nephropathy.^6^

Recently several studies have shown that treatment with Angiotensin-Converting Enzyme (ACE) inhibitors or Angiotensin Receptor Antagonists can help in reducing the symptoms of diabetic nephropathy by virtue of partial reduction in proteinuria and attenuates the progression of chronic kidney disease (CKD) to end-stage kidney disease.^7^ However, it has also been shown that many patients do not respond to these agents and they progress to end-stage renal disease at an early stage.^8^ More recent studies have reported that dietary antioxidants seem to be a potential adjuvant therapy to prevent or delay diabetic complication such as nephropathy.^9^^,^^10^

2, 3 Dimethoxy-5 methyl-6-decaprenyl benzoquinone, i.e., coenzyme Q10 or ubiquinone is a vitamin-like substance which is lipid soluble in nature and hydrophobic interior of the phospholipid bilayer of cell membrane. It exists in wide range of dietary items including meat, fish, vegetable oils, and nuts.^11^ It has been previously reported that coenzyme Q10 has a potent anti-inflammatory,^12^ antihypertensive,^13^ antioxidant^14^ and antidiabetic^11^ activities.

It was also reported that dipeptidyl peptidase-IV (DPP-IV) inhibitors, as sitagliptin is a new class of anti-diabetic drug that improve glycemic control by stimulating the incretin axis. Sitagliptin may play a role in its renoprotective effect by modulating several oxidative stress markers and histopathological alteration confirmed that sitagliptin administration prevented kidney damage, which provided structural support for the renal shielding effect.^15^^,^^16^ Therefore, it was thought to combine antioxidant like coenzyme Q10 and sitagliptin to study their renoprotective effect in experimentally induced nephropathy. Hence, the present study was aimed to investigate the protective effect of coenzyme Q10 alone and its combination with sitagliptin on STZ-nicotinamide-induced diabetic nephropathy.

## Materials and methods

### Drugs and chemicals

Sitagliptin and coenzyme Q10 were obtained from Zydus Cadila, Ahmedabad, India. Streptozotocin and nicotinamide were purchased from Himedia (Mumbai, India). Kits used in the study were procured from standard company. All other chemicals and reagents used in the study were of analytical grade.

### Experimental animals

The experimental protocol was approved by the Institutional Animal Ethics Committee and carried out in accordance with CPCSEA (Committee for the Purpose of Control and Supervision of Experiment on Animal) guidelines. The experiment was carried out on healthy adult Wistar rats weighing 200–250 g of either sex. Rats were housed in polypropylene cages, maintained under standardized condition (12-h light/dark cycle, 24 °C, 35 to 60% humidity) and allowed free access to diet (Nav Maharashtra Oil Mills Pvt. Ltd., Pune) and purified drinking water *ad libitium*.

### Induction of diabetic nephropathy

Type 2 diabetes was induced in overnight fasted adult albino wistar rats (200–250 g) by a single intraperitoneal (i.p.) injection of 65 mg/kg streptozotocin (dissolved in citrate buffer, pH 4.5), followed by the i.p. administration of 110 mg/kg of nicotinamide (dissolved in normal saline).^17^^,^^18^ Hyperglycemia was confirmed by elevated blood glucose levels at 72 h and then on day 7 after injection. Those animals with fasting blood glucose level greater than 200 mg/dl were considered as diabetic and were used for diabetic nephropathy studies.

### Experimental design

Diabetic rats were randomly divided into five groups each consisting of six animals.

Group I: Normal control rats (distilled water10ml/kg, p.o.).
Group II: Diabetic control rats.
Group III: Diabetic rats treated with 10 mg/kg coenzyme Q10 (1% aqueous solution of Tween 80, i.p.).^19^
Group IV: Diabetic rats treated with sitagliptin (10 mg/kg, p.o).^20^
Group V: Diabetic rats treated with the combination of coenzyme Q10 (10 mg/kg) and sitagliptin (10 mg/kg)

All the aforementioned treatments were given daily to the respective group of animals for 42 days.

At the end of the experiments, blood samples were collected from the retro orbital plexus of rats under light ether anesthesia, using glass capillaries and stored with or without disodium ethylene diamine tetra-acetate for estimation of biochemical parameter. For separation of serum, blood was allowed to clot for 15 min and it was then centrifuged at 5000 rpm for 20 min. The serum was stored at −20 °C until further biochemical estimation.

Glycated hemoglobin (Hb1_AC_) was estimated using whole blood. Creatinine, urea, uric acid, total cholesterol, triglyceride and HDL-C were estimated from serum using standard diagnostic kit (SPAN Diagnostics, India). Rats were kept in metabolic cages for 24 h for urine collection. Urine samples were centrifuged at 1400 rpm for 5 min after proper dilution and the supernatant was collected to determine urinary micro protein level using standard kit.

### Estimation of biomarkers of oxidative stress

Kidney was removed and kept on autoclaved inverted Petridish in cold condition with ice cubes. The tissues were cross chopped with surgical scalpel into fine slices in chilled 0.25 M sucrose, quickly blotted on a filter paper. They were minced and homogenized in 10 mM Tris-HCl buffer, pH 7.4 (10%w/v) with 25 strokes of tight Teflon pestle of glass homogenizer at a speed of 10,000 ×g at 0 C using the Remi cooling centrifuge. The clear supernatant obtained was used for assay of lipid peroxidation (MDA content),^21^ endogenous antiperoxidative enzymes such as superoxide dismutase (SOD),^22^ catalase^23^ and GSH.^24^

### Estimation of myeloperoxidase (MPO) activity

Myeloperoxidase (MPO) activity in kidney tissue was determined.^25^ The kidney tissue was homogenized in 0.5% hexadecyltrimethyammonium bromide containing 50 mM potassium phosphate buffer (pH 6) using a polytron tissue homogenizer. After freeze-thawing three times, the samples were centrifuged at 20,000 x *g* for 15 min at 40 °C, and the resulting supernatant was assayed spectrophotometrically for MPO activity. In brief, 0.1 ml of sample was mixed with 2.9 ml of 50 mM potassium phosphate buffer (pH 6) containing 0.167 mg/ml O-dianisidine dihydrochlorde and 0.0005% hydrogen peroxide. The change in absorbance at 460 nm was then measured for 5 min using spectrophotometer. Myeloperoxidase activity data are presented as U/g tissue.

### Determination of TNF-α and TGF-β by ELISA

Tumor necrosis factor alpha (TNF-α) and Transforming growth factor beta (TGF-β) levels in homogenized kidney tissues were determined by quantitative enzyme-linked immunosorbent assay (ELISA) kits according to the manufacturer’s instructions [Rat TNF-α, catalog no. SEA133RA & TGF-β, catalog no. SEA124RA kits were purchased from USCN Life Science Inc.].

### Estimation of tissue nitrite content

Nitrite was estimated colorimetrically with the Griess reagent in protein free supernatant of kidney homogenate.^26^ Equal volumes of protein free supernatant of kidney homogenate and Griess reagent (sulfanilamide 1%w/v, naphthylenediamine dihydrochlorde 0.1% w/v and orthophosphoric acid 2.5% v/v) were mixed and incubated at room temperature for 10 min and the absorbance was determined at 540 nm wavelength and compared to those of known concentrations of sodium nitrite.

### Histopathology

After sacrifice, kidney tissues of each group was rapidly dissected out and washed immediately with saline and fixed in 10% phosphate buffered formalin. Paraffin-embedded specimens were cut into 5 μm-thick sections and stained with hematoxylin and eosin (H&E). The sections were examined under the light microscope (Olympus BX10, Tokyo, Japan) for the presence of histopathological changes and photomicrographs (Olympus DP12 camera, Japan) were taken. The observer performing histopathological evaluation was blinded to the animal treatment group.

### Statistical analysis

All the data are expressed as mean ± SEM. Statistical significance between more than two groups was tested using one-way ANOVA followed by the Bonferroni multiple comparisons test as appropriate using computer based fitting program (Prism, GraphPad version 5, GraphPad Software, Inc). The significance level was set at *p* < .05 for all tests.

## Results

### Effect of coenzyme Q10, sitagliptin or concomitant administration on body weight and kidney weight

We did not observe any mortality among the experimental groups. The body weight of the diabetic rats showed a significant decrease (*p* < .01) after the administration of STZ-nicotinamide as compared to normal control rats. The treatment with coenzyme Q10 or sitagliptin or coenzyme Q10 + sitagliptin did not show any significant reduction in the body weight as compared with diabetic control rats (Table 1).

**Table 1.** Effect of coenzyme Q10, sitagliptin or combination of both on body weight and kidney weight.

Groups	Initial body weight (g)	Final body weight (g)	Kidney weight (g)
Normal control (a)	235.0 ± 5.28	255.0 ± 5.21	0.59 ± 0.12
Diabetic control (b)	271.7 ± 9.14	186.7 ± 11.5###	1.18 ± 0.06###
Coenzyme Q10 (10 mg/kg) (c)	225.0 ± 8.76	196.7 ± 9.28	0.94 ± 0.02***
Sitagliptin (10 mg/kg) (d)	218.0 ± 8.63	204.0 ± 9.55	0.83 ± 0.02***
Coenzyme Q10 + Sitagliptin (e)	229.7 ± 12.09	212.7 ± 10.38	0.76 ± 0.01***+

Values are expressed as mean ± SEM; *n* = 6.

a vs. b, ###*p* < .001;

b vs. c, b vs. d and b vs. e, ***p < .001; c vs. e, +*p* < .05.

There was a significant (*p* < .001) increase in kidney weight after 6th week in diabetic control rats as compared to normal control rats, while the treatment with coenzyme Q10 or sitagliptin or coenzyme Q10 + sitagliptin showed a significant (*p* < .001) reduction in kidney weight as compared to diabetic control rats. Moreover, co-administration of coenzyme Q10 with sitagliptin showed more beneficial effect in reducing kidney weight than when coenzyme Q10 administered singly (Table 1).

### Effect of coenzyme Q10, sitagliptin or combination of both on urine volume and urinary protein

**Figure 1.**
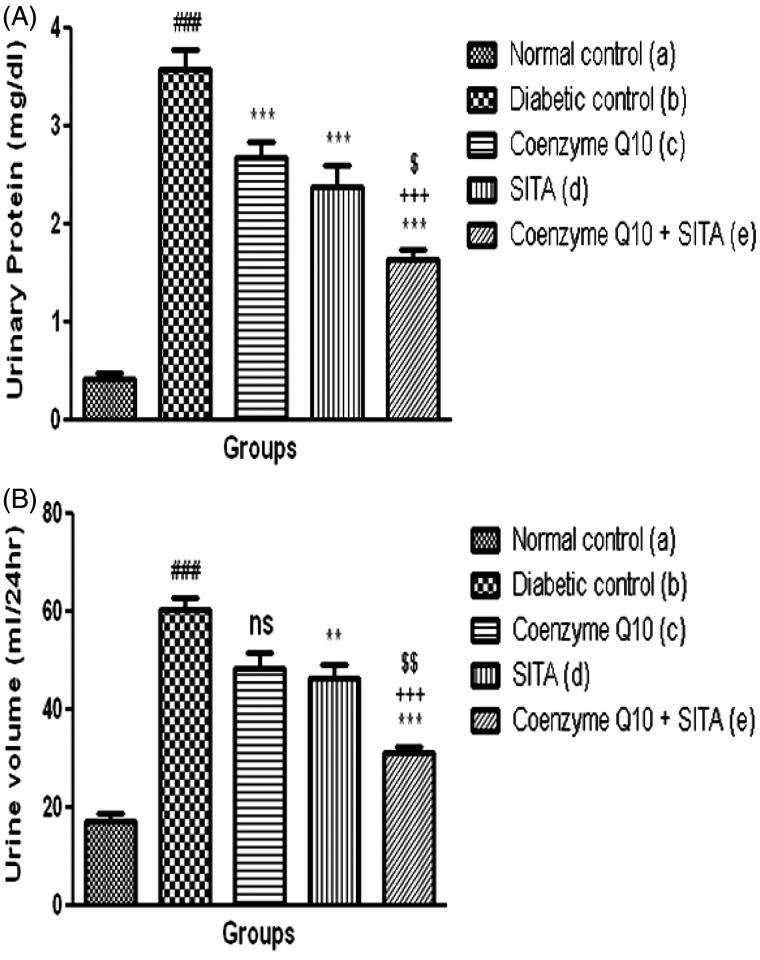
Effect of coenzyme Q10, sitagliptin or combination of both on (A) urinary protein and (B) urine volume. Values are expressed as mean ± SEM; *n* = 6; a vs. b, ###*p* < .001; b vs. c, b vs. d and b vs. e, **p < .01, ***p < .001; c vs. e, +++*p* < .001; d vs. e, $*p* < .05, $$*p* < .01.

Six week post STZ-nicotinamide injection caused a significant (*p* < .001) increase in urinary protein of diabetic rats as compared to normal control rats. The treatment with coenzyme Q10, sitagliptin or coenzyme Q10 + sitagliptin showed a significant (*p* < .001) reduction in urinary protein when compared to diabetic control rats (Figure 1(A)).

In diabetic control group, urine volume was significantly (*p* < .001) increased when compared to the normal control rats. When diabetic rats treated with sitagliptin or coenzyme Q10 + sitagliptin there was a significant (*p* < .01; *p* < .001) reduction in urine volume as compared to diabetic control rats, while the treatment with coenzyme Q10 did not show a significant reduction in urine volume as compared to diabetic rats (Figure 1(B)).

### Effect of coenzyme Q10, sitagliptin or concomitant administration on glycated hemoglobin level

**Figure 2.**
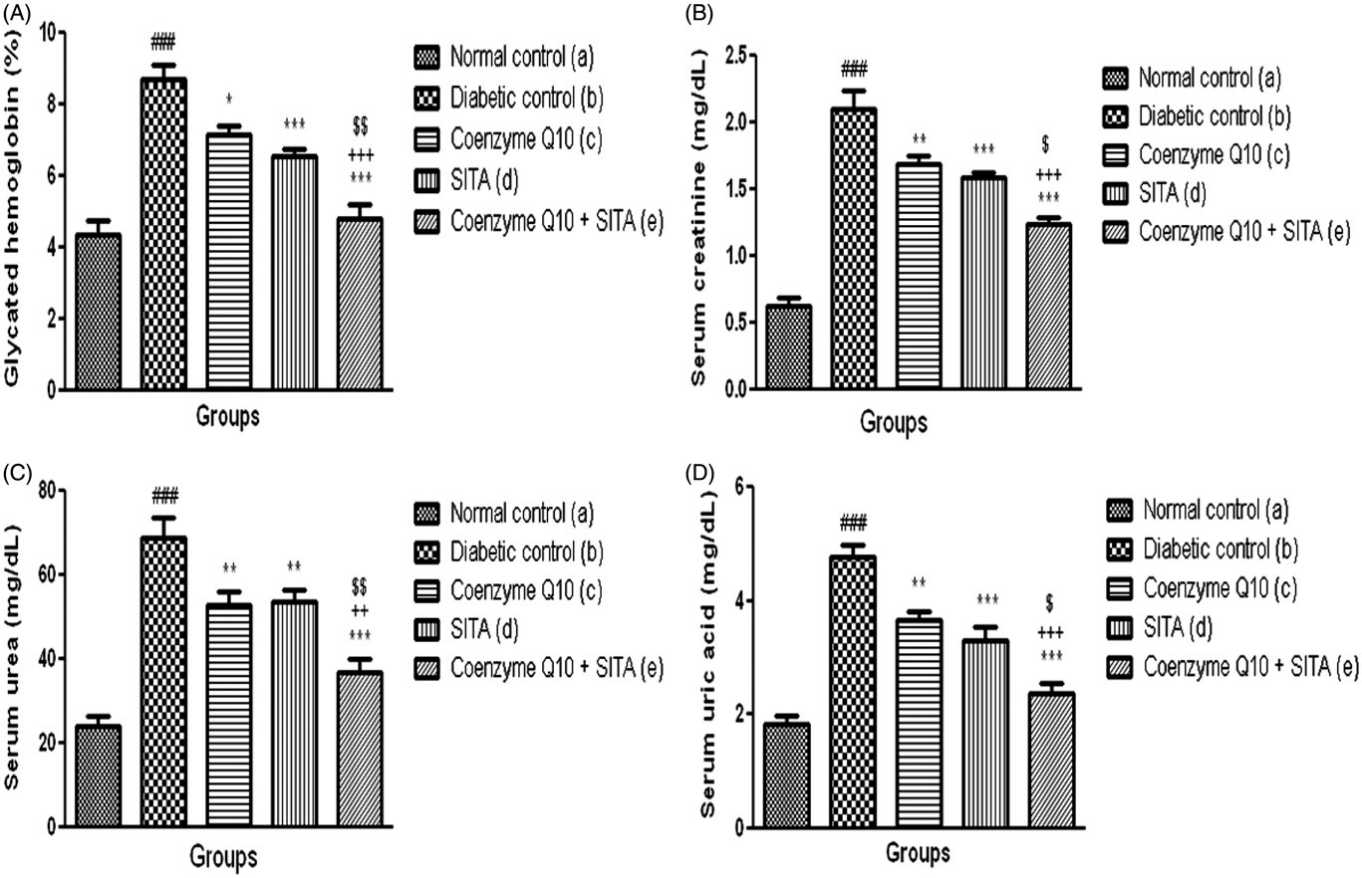
Effect of coenzyme Q10, sitagliptin or combination of both on (A) glycated hemoglobin (B) serum creatinine (C) serum urea and (D) serum uric acid. Values are expressed as mean ± SEM; *n* = 6; a vs. b, ###*p* < .001; b vs. c, b vs. d and b vs. e, *p < .05, **p < .01, ***p < .001; c vs. e, ++*p* < 0.01, +++*p* < 0.001; d vs. e, $*p* < .05, $$*p* < .01.

In the diabetic control rats, glycated hemoglobin level was significantly (*p* < .001) increased when compared to normal control rats. The diabetic rats treated with coenzyme Q10 showed a significant (*p* < .05) reduction in glycated hemoglobin level as compared to diabetic control rats. However, the treatment with sitagliptin or coenzyme Q10 + sitagliptin showed a significant (*p* < .001) reduction in glycated hemoglobin level as compared to diabetic control rats. Moreover, co-administration of coenzyme Q10 with sitagliptin showed more beneficial effect in reducing glycated hemoglobin level than when coenzyme Q10 or sitagliptin administered singly (Figure 2(A)).

### Effect of coenzyme Q10, sitagliptin or combination of both on serum creatinine, urea and uric acid

STZ-nicotinamide injection caused a marked reduction in renal function characterized by significant (*p* < .001) increase in serum creatinine, urea and uric acid levels as compared to normal control rats. Thus, these data indicate that single i.p injection of STZ-nicotinamide impairs kidney functions. Treatment with coenzyme Q10, sitagliptin or coenzyme Q10 + sitagliptin showed a significant (*p* < .01; *p* < .001 and *p* < .001) reduction in serum creatinine levels as compared to diabetic control rats. In contrast, the combination of coenzyme Q10 and sitagliptin showed more beneficial effect in reducing serum creatinine levels than that of mono-therapy (coenzyme Q10 or sitagliptin) (Figure 2(B)).

The treatment with coenzyme Q10, sitagliptin or coenzyme Q10 + sitagliptin showed a significant (*p* < .01; *p* < .01 and *p* < .001) reduction in urea levels as compared to diabetic control rats. Treatment with coenzyme Q10, sitagliptin or coenzyme Q10 + sitagliptin showed a significant (*p* < .01; *p* < .001 and *p* < .001) decrease in uric acid levels as compared to diabetic control rats, respectively. In addition, concomitant administration of coenzyme Q10 and sitagliptin showed more beneficial effect in reducing serum urea and uric acid levels than coenzyme Q10 or sitagliptin given alone (Figure 2(C,D)).

### Effect of coenzyme Q10, sitagliptin or combination of both on lipid profiles

**Figure 3.**
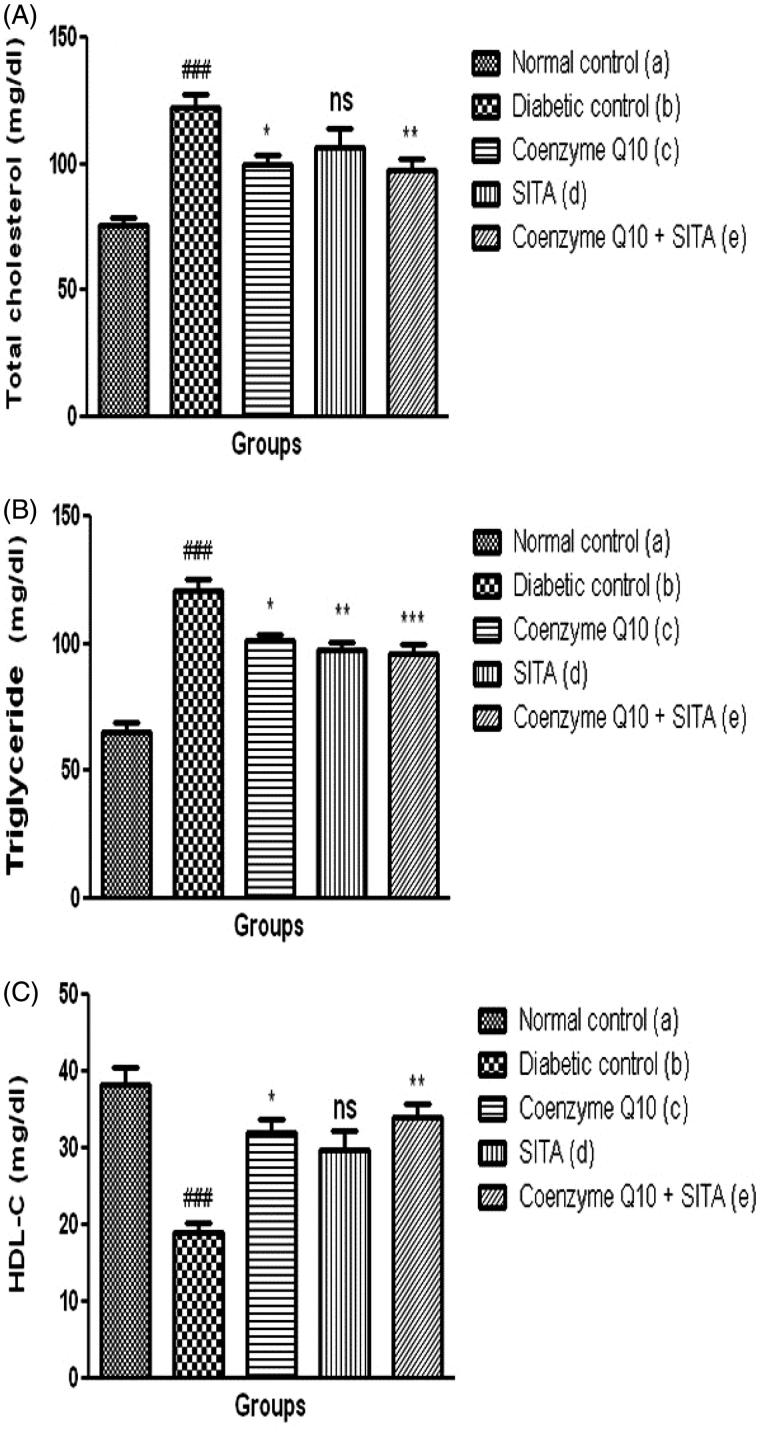
Effect of coenzyme Q10, sitagliptin or combination of both on (A) total cholesterol (B) triglyceride and (C) HDL-C. Values are expressed as mean ± SEM; *n* = 6; a vs. b, ###*p* < .001; b vs. c, b vs. d and b vs. e, *p < .05, **p < .01, ***p < .001.

STZ-nicotinamide injection showed a significant (*p* < .001) increase in serum total cholesterol, serum triglyceride and a decrease in HDL-C levels as compared to normal control rats. Treatment with coenzyme Q10 showed a significant (*p* < .05) reduction in serum total cholesterol, serum triglyceride and an increase in HDL-C levels as compared to diabetic control rats. Moreover, the treatment with coenzyme Q10 + sitagliptin showed a more significant reduction in serum total cholesterol (*p* < .01), serum triglyceride (*p* < .001) and a rise in HDL-C (*p* < .01) levels as compared to diabetic control rats, while the sitagliptin-treated rats did not show a significant difference in serum total cholesterol and HDL-C levels as compared to diabetic control rats. There was a significant improvement on the lipid profile in combination therapy (coenzyme Q10 and sitagliptin) than that of mono-therapy (coenzyme Q10 or sitagliptin) (Figure 3(A–C)).

### Effect of coenzyme Q10, sitagliptin or combination of both on markers of oxidative stress in renal tissue

The content of MDA, end product of lipid peroxidation and marker of oxidative stress was significantly (*p* < .001) increased in renal tissue of diabetic control rats as compared to non-diabetic rats after six weeks of study. There was a significant (*p* < .001) decrease in the levels of GSH, an endogenous antioxidant and antiperoxidative enzymes (SOD and catalase) in renal tissue as compared to normal control group.

**Figure 4.**
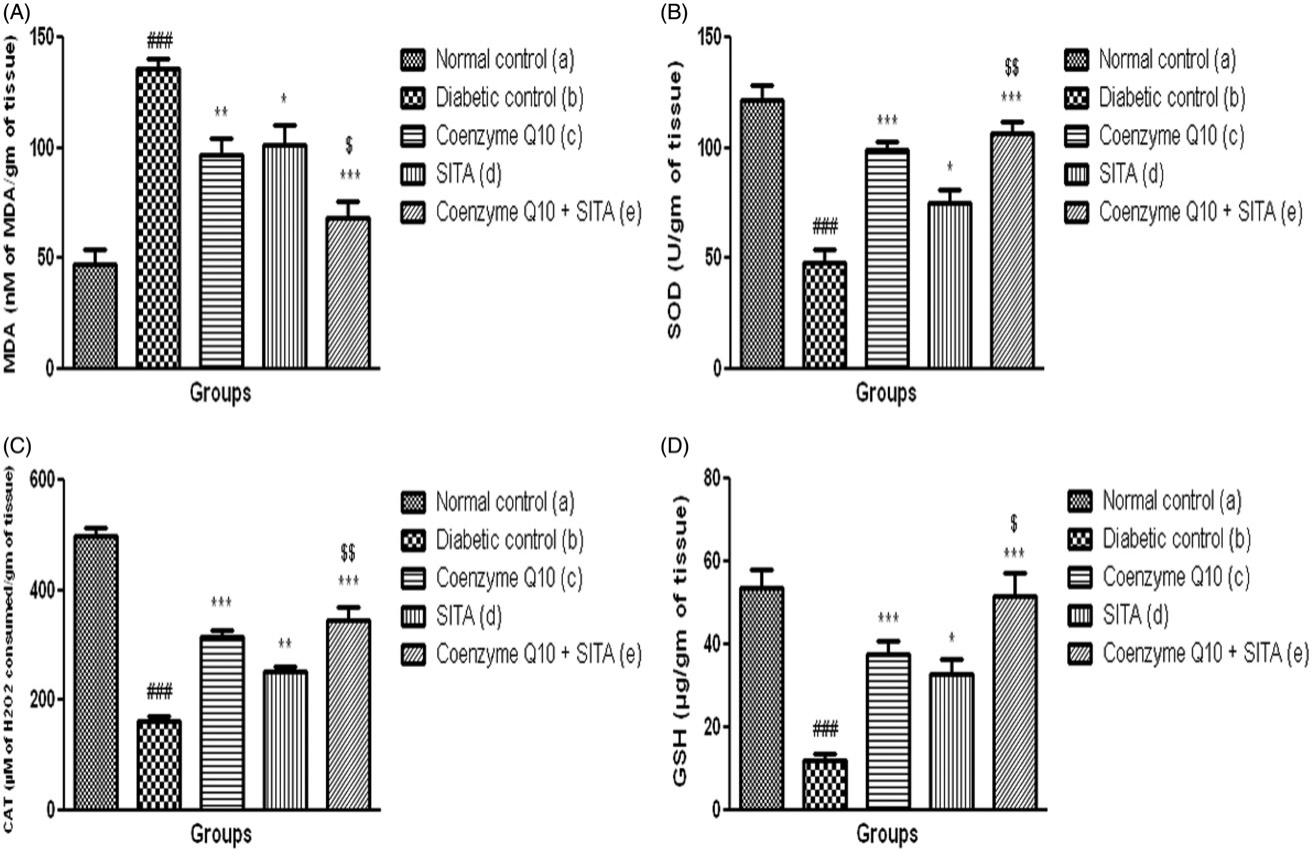
Effect of coenzyme Q10, sitagliptin or combination of both on (A) MDA (B) SOD (C) Catalase and (D) GSH. Values are expressed as mean ± SEM; *n* = 6; a vs. b, ###*p* < .001; b vs. c, b vs. d and b vs. e, *p < .05, **p < .01, ***p < .001; d vs. e, $*p* < .05, $$*p* < .01.

The treatment of diabetic rats with coenzyme Q10 or sitagliptin or coenzyme Q10 + sitagliptin showed a significant decrease in the levels of MDA (*p* < .01; *p* < .05 and *p* < .001) and GSH (*p* < .001; *p* < .05 and *p* < .001) as compared to diabetic control rats. Coenzyme Q10 or coenzyme Q10 + sitagliptin showed a significant (*p* < .001) increase in SOD and catalase activities, while the sitagliptin treated rats showed a significant (*p* < .05; *p* < .01) increase in SOD and catalase activities. There was more significant alteration in MDA, SOD, catalase and GSH levels when coenzyme Q10 and sitagliptin were given together (Figure 4(A–D)).

### Effect of coenzyme Q10, sitagliptin or combination of both on TNF-α, myeloperoxidase (MPO) activity, TGF-beta and nitrite content in renal tissue

**Figure 5.**
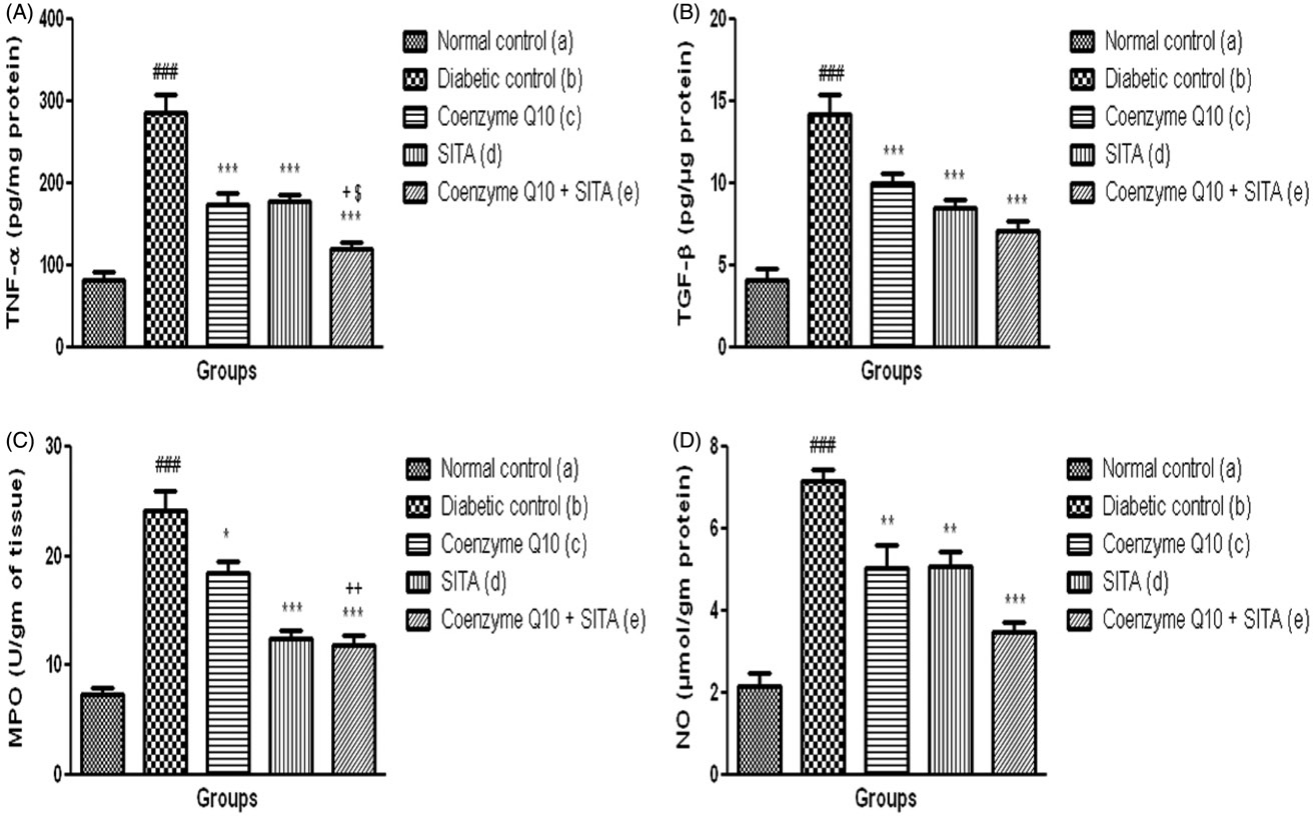
Effect of coenzyme Q10, sitagliptin or combination of both on (A) TNF-α (B) TGF-β (C) MPO and (D) NO. Values are expressed as mean ± SEM; *n* = 6; a vs. b, ###*p* < .001; b vs. c, b vs. d and b vs. e, *p < .05, **p < .01, ***p < .001; c vs. e +*p* < .05, ++*p* < .01; d vs. e, $*p* < .05.

Diabetic control rats showed a significant (*p* < .001) increase in inflammatory markers such as renal TNF-α level, MPO activity and TGF-β as compared to normal control rats. The treatment with coenzyme Q10 in STZ-nicotinamide treated rats showed a significant reduction in TNF-α (*p* < .001) level, MPO activity (*p* < .05) and TGF-β (*p* < .001) in renal tissue when compared to diabetic control rats. However, treatment with coenzyme Q10 + sitagliptin showed more significant (*p* < .001) decrease in TNF-α level, MPO activity and TGF-β in renal tissue as compared to diabetic rats treated with sitagliptin or coenzyme Q10 alone. There were more significant changes in TNF-α, MPO and TGF-β levels when coenzyme Q10 and sitagliptin were given together than that of coenzyme Q10 or sitagliptin administered individually (Figure 5(A–C)).

Nitrite content was significantly (*p* < .001) increased in renal tissue of diabetic rats as compared to normal control group. The coenzyme Q10 + sitagliptin treatment group showed more significant (*p* < .001) decrease in renal nitrite content as compared to diabetic untreated group, while coenzyme Q10 or sitagliptin alone caused a significant (*p* < .01) decrease in nitrite content in renal tissue as compared to diabetic rats, but this effect was lesser than the combination therapy (Figure 5(D)).

### Histopathological studies

**Figure 6.**
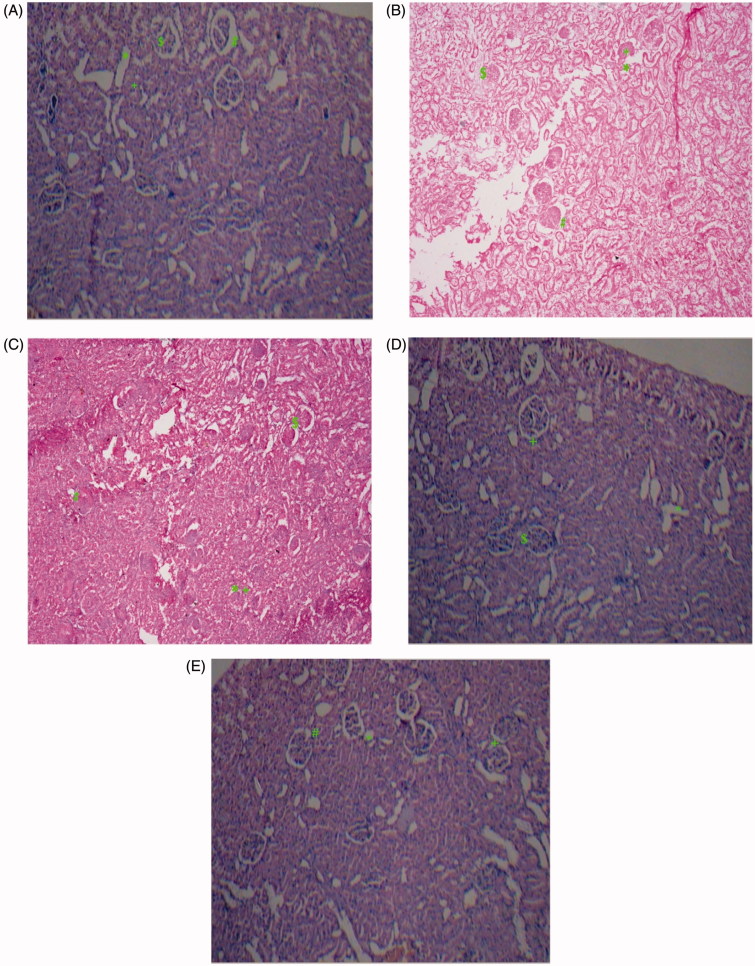
Light microscopy of kidney tissues from rats (HE stained kidney sections). (A) Control group, (B) Diabetic control group, (C) Coenzyme Q10 (D) Sitagliptin (E) Coenzyme Q10 + Sitagliptin.

The architecture of the kidney was disturbed with diabetic control rats as compared to normal structural features of control animal. In the normal control group, the histopathological examination of kidney tissue showed normal appearance of glomerulli and tubules. Renal tissue section of diabetic rats showed glomerulosclerosis ($), tubular vacuolization (*), interstitial fibrosis (+) and thickening of glomerular basement membrane (#). The treatment with coenzyme Q10 or sitagliptin showed mild glomerular necrosis, interstitial fibrosis, moderate tubular vacuolization, thickening of glomerular basement membrane. However, the treatment with concomitant administration of coenzyme Q10 with sitagliptin showed mild glomerular fibrosis, tubular swelling and interstitial fibrosis but with absence of glomerular necrosis (Figure 6(A–E)).

## Discussion

Diabetic nephropathy (DN) or nephropatia deabetica or Kimmelstiel–Wilson syndrome or nodular diabetic glomerulosclerois in type 2 diabetes is the most common cause of end-stage renal disease and one of the leading causes of morbidity and mortality worldwide.^27^ There is a great challenge in biomedical research for the treatment of complications due to diabetes. In diabetic nephropathy there are always increased levels of serum glucose, creatinine, urea and uric acid which are the markers of DN.^28^ Recent reports have shown that some of the antioxidants such as coenzyme Q10, vitamin C and lipoic acid are beneficial in decreasing the elevated hemoglobin A_1C_, urea and creatinine levels in alloxan-induced diabetic rats.^29^ In the present study co-administration of coenzyme Q10 and sitagliptin caused a significant reduction in elevated hemoglobin A_1C_, urea, creatinine and uric acid levels as compared to diabetic rats. These results are in accordance with the earlier study in which it was shown that sitagliptin alone produced a beneficial effect on diabetic nephropathy.^16^ In this study sitagliptin when administered along with an antioxidant like coenzyme Q10 produced a synergic effect in reducing the development of DN.

Recently, several studies have shown that oxidative stress is responsible for the pathogenesis of diabetic injuries. Free radicals such as superoxide and lipid peroxidation product like malondialdehyde can induce cell and tissue damage.^30^^,^^31^ It was reported that some antioxidants such as coenzyme Q10, vitamin E, and antiperoxidative such as SOD, CAT, and GSH protect the cells and tissue against oxidative stress-mediated injuries.^32^ Results in the present study also indicate that there is an increase in the oxidative stress after STZ-nicotinamide induced diabetic nephropathy. In the present study, there were significant reduction in lipid peroxidation and an increase in SOD, CAT, and GSH levels after the treatment with coenzyme Q10 or sitagliptin or coenzyme Q10 + sitagliptin. In contrast, co-administration of coenzyme and sitagliptin has shown more significant alteration in MDA, SOD, catalase and GSH levels than when administered alone.

DN occurs as a result of the effects of both metabolic and hemodynamic insults, which at cellular level lead to the activation of intracellular signaling pathway and transcription factors. This effect is due to the release of TNF-α, myeloperoxidase (MPO) and TGF-β.^33^^,^^34^ In the present study, administration of coenzyme Q10 or sitagliptin or coenzyme Q10 + sitagliptin resulted in decrease in renal TNF-α, TGF-β and MPO levels as compared to diabetic control rats. However, the treatment with coenzyme Q10 + sitagliptin showed more renoprotective effect by virtue of significant reduction of TNF-α, TGF-β and MPO levels in renal tissues. It was reported that level of nitrite, an oxidized end product of NO, which might be attributed to formation of peroxynitrite by reaction of NO with generated superoxide radicals.^35^ In the present study, diabetic rats showed a significant increase in tissue nitrite content as compared to normal control rats, while the treatment with coenzyme Q10 or sitagliptin or coenzyme Q10 + sitagliptin restored the levels of renal nitrite.

## Conclusion

These results indicate that treatment with coenzyme Q10 or sitagliptin showed significant renoprotective effects against STZ-nicotinamide-induced diabetic nephropathy. However, co-administration of coenzyme Q10 and sitagliptin showed a better renoprotective effect than coenzyme Q10 or sitagliptin alone by virtue of amelioration of lipid peroxidation as well as due to improvement of renal function and suppression of TNF-α, TGF-β, MPO activity and nitrite content in renal tissue. It was concluded that adjuvant therapy of coenzyme Q10 with sitagliptin might delay or prevent diabetic nephropathy.
